# Prognostic value of albumin to globulin ratio in non-muscle-invasive bladder cancer

**DOI:** 10.1007/s00345-020-03586-1

**Published:** 2021-01-26

**Authors:** Fahad Quhal, Benjamin Pradere, Ekaterina Laukhtina, Reza Sari Motlagh, Hadi Mostafaei, Keiichiro Mori, Victor M. Schuettfort, Pierre I. Karakiewicz, Morgan Rouprêt, Dmitry Enikeev, Michael Rink, Mohammad Abufaraj, Shahrokh F. Shariat

**Affiliations:** 1 Department of Urology, Comprehensive Cancer Center, Medical University of Vienna, Vienna, Austria; 2 Department of Urology, King Fahad Specialist Hospital, Dammam, Saudi Arabia; 3 Department of Urology, University Hospital of Tours, Tours, France; 4 Institute for Urology and Reproductive Health, Sechenov University, Moscow, Russia; 5 Research Center for Evidence Based Medicine, Tabriz University of Medical Sciences, Tabriz, Iran; 6 Department of Urology, Jikei University School of Medicine, Tokyo, Japan; 7 Department of Urology, University Medical Center Hamburg-Eppendorf, Hamburg, Germany; 8 Cancer Prognostics and Health Outcomes Unit, Division of Urology, University of Montreal Health Center, Montreal, Canada; 9 Predictive onco-uro, AP-HP, Urology Hôpital Pitié-Salpêtrière, Sorbonne Université, GRC n°5, 75013 Paris, France; 10 Division of Urology, Department of Special Surgery, Jordan University Hospital, The University of Jordan, Amman, Jordan; 11 Department of Urology, Weill Cornell Medical College, New York, NY USA; 12 Department of Urology, University of Texas Southwestern, Dallas, TX USA; 13 Department of Urology, Second Faculty of Medicine, Charles University, Prague, Czech Republic; 14 European Association of Urology Research Foundation, Arnhem, Netherlands; 15 Karl Landsteiner Institute, Wahringer Gurtel 18-20, 1090 Vienna, Austria

**Keywords:** Non-muscle-invasive, Bladder cancer, Progression, AGR, Albumin, Globulin, BCG

## Abstract

**Purpose:**

To investigate the prognostic value of preoperative serum albumin to globulin ratio (AGR) in patients with non-muscle-invasive bladder cancer (NMIBC) treated with transurethral resection of bladder tumor (TURB) with or without intravesical therapy (IVT).

**Materials and methods:**

We retrospectively reviewed 1,096 consecutive patients with NMIBC. Levels of albumin and globulin were obtained before TURB and used to calculate the preoperative AGR level. Multivariable Cox regression analyses were performed to assess the prognostic effect of preoperative AGR on oncologic outcomes. Subgroup analyses were performed in patients based on the European Association of Urology (EAU) risk groups for NMIBC.

**Results:**

Low AGR levels were observed in 389 (35.5%) patients. The median follow-up was 63.7 months (IQR 25.3–111). On multivariable Cox regression analysis, low AGR was associated with increased risk of progression to muscle-invasive BCa (MIBC) (HR 1.81, 95% CI 1.22–2.68, *P* = 0.003). The addition of AGR only minimally improved the discrimination ability of a base model that included established clinicopathologic features (C-index = 0.7354 vs. C-index = 0.7162). Low preoperative AGR was not significantly associated with the risk of disease recurrence (*P* = 0.31). In subgroup analyses based on patients’ EAU risk groups, low preoperative AGR was not associated with recurrence-free survival (RFS) (*P* = 0.59) or progression-free survival (PFS) (*P* = 0.22) in any of the risk groups. Additionally, in patients treated with Bacillus Calmette–Guerin (BCG) for intermediate- or high-risk NMIBC, low AGR failed to predict disease recurrence or progression.

**Conclusion:**

Preoperative serum AGR levels independently predicted the risk of disease progression in patients with NMIBC. However, it was not found to be associated with either RFS or PFS in NMIBC patients based on their EAU risk group. This marker seems to have a limited role in NMIBC at the present time. However, further research is needed to investigate this marker in combination with other systemic inflammatory markers to help improve prediction in this heterogeneous group of patients.

**Supplementary Information:**

The online version contains supplementary material available at 10.1007/s00345-020-03586-1.

## Introduction

Bladder cancer (BCa) is the 9th most commonly diagnosed cancer, and the 13th cause of cancer-related mortality worldwide [1]. Approximately 70–80% of BCa patients present initially as non-muscle-invasive (NMIBC) [2]. The standard treatment for those patients is transurethral resection (TURB) followed by intravesical therapy (IVT) depending on the patient’s risk. However, despite treatment, around 50% experience disease recurrence and about 10–20% experience disease progression to muscle-invasive disease (MIBC) [2–4].

Patients with MIBC who experience disease progression from primary NMIBC have worse prognosis compared to those who present with primary MIBC, with up to 50% succumbing to their disease despite radical cystectomy [5, 6]. Identifying those patients before disease progression is of utmost importance, as it may improve their outcomes by adapting treatment strategy [7–10]. The current risk prediction tools like the European Organization for Research and Treatment of Cancer (EORTC) or the Spanish Urological Club for Oncological Treatment (CUETO) scoring models rely on clinicopathologic features. While these prediction tools have improved the management of NMIBC, validation studies have reported their limited accuracy, especially in high-risk groups [4]. Identifying additional accurate biomarkers can potentially help improve the predictive ability of these models, to help tailor the treatment for those at higher risk of progression [11–15].

Albumin and globulin are major components of serum proteins. These proteins play an essential role in immunity and inflammation. Albumin-to-globulin ratio (AGR) has been reported to be a potential prognostic biomarker in several cancers [16, 17].

We hypothesized that low preoperative AGR is associated with worse oncologic outcomes in NMIBC patients. To test this theory, we studied the association between preoperative AGR and the risk of disease recurrence and progression in a large multicentric cohort of NMIBC patients. We also tested the prognostic value of preoperative AGR within each EAU risk group [2].

## Materials and methods

### Patient population and treatment

This study was approved by the institutional review boards of the participating centers. Waiver of individual informed consent was granted for this retrospective study. We retrospectively reviewed the medical records of 1,096 consecutive patients with NMIBC treated with TURB. Immediate single-dose postoperative instillation chemotherapy, adjuvant intravesical chemotherapy, or adjuvant Bacillus Calmette–Guérin (BCG) immunotherapy were administered according to patient’s risk group, guideline treatment recommendations and patient-physician shared decision-making. Repeat TURB was not routinely performed. All specimens were staged and graded according to the 2009 TNM classification and the 1973 World Health Organization system, respectively, by genitourinary pathologists. Serum albumin and globulin were collected within 30 days before TURB. No known systematic inflammatory disease or urinary tract infection was seen in all patients. Patients were assigned into low-, intermediate-, and high-risk groups of NMIBC, according to the European Association of Urology (EAU) guidelines [2].

### Follow-up

The postoperative follow-up included physical examination, urine cytology, and cystoscopy scheduled generally at every 3 months for the first 2 years, every 6 months for the three following years, and then yearly. Imaging of the upper urinary tract was performed based on pathologic features according to guidelines and at physician discretion. Disease recurrence was defined as the first pathologically proven tumor relapse of any stage or grade, whereas disease progression was defined as muscle-invasive tumor [18].

### Finding the optimal cutoff value for preoperative AGR

The preoperative AGR cut-off point was determined by Receiver Operating Characteristics (ROC) curve analysis using Youden index [19]. For the whole cohort, median preoperative AGR was 1.54 (1.38–1.69); the optimal cut-off value corresponding to the maximum joint sensitivity and specificity was 1.41. Based on this cutoff value, a total of 389 patients (35.5%) were detected with a preoperative AGR < 1.41 ‘low preoperative AGR’, whereas 797 patients (64.5%) had an AGR ≥ 1.41 ‘normal preoperative AGR’.

### Statistical analysis

Comparisons of study groups were assessed by Chi-square and Mann–Whitney *U* tests. Kaplan–Meier curves and the log-rank test were used to estimate and determine the statistical differences between study groups. Univariable and multivariable Cox regression analyses were used to test the association between preoperative AGR and oncologic outcomes. Exploratory subgroup analyses were performed in patients based on the EAU risk groups, and BCG-treated high-risk patients. Results were considered significant if two-sided *P*-value was < 0.05. Data analyses were performed using STATA 16 (Stata Corp., College Station, TX).

## Results

### Association with clinical and pathologic features

The clinicopathologic features of 1,096 patients with NMIBC treated with TURB are stratified by preoperative AGR level and summarized in Table 1. The median follow-up was 63.7 months (IQR 25.3–111). The median age for the overall cohort was 67 years (IQR 58–74). There were no statistically significant differences in age, gender, grade, stage, and tumor size or number between the low and normal preoperative AGR groups. There were more patients with concomitant carcinoma in-situ (CIS) in the low AGR group.

**Table 1:** Clinicopathologic features of 1096 patients with non-muscle-invasive bladder cancer, stratified by preoperative serum albumin-to-globulin ratio (AGR)

Variables	Total	Normal AGR	Low AGR	*P*-value
Number of patients, *n* (%)	1096	797 (64.5)	389 (35.5)	
Median age (IQR)	67 (58–74)	65.5 (57.9–74)	67.7 (60–75)	0.11
Gender, *n* (%)				
Female	254 (23.2)	158 (62.2)	96 (37.8)	0.38
Male	842 (76.8)	549 (65.2)	293 (34.8)	
Smoking status, *n* (%)				
Never smoked	267	160 (59.93)	107 (40.1)	0.20
Former smoker	322	213 (66.2)	109 (33.8)	
Current smoker	507	334 (65.9)	173 (34.1)	
Prior recurrent rate, *n* (%)				
Primary	916	600 (65.5)	316 (34.5)	0.69
≤ 1 recurrent/y	88	51(51.9)	37(42.1)	
> 1 recurrent/y	92	56(60.9)	36 (39.1)	
Pathologic T stage, *n* (%)				
pTa	653	419 (64.2)	234 (35.8)	0.77
pT1	443	288 (65)	155 (35)	
Pathologic tumor grade, *n* (%)				
Grade 1	230	159 (69.1)	71 (30.9)	0.21
Grade 2	383	238 (62.1)	145 (37.9)	
Grade 3	483	310 (64.2)	173 (35.8)	
Concomitant CIS, *n* (%)	47	23 (48.9)	24 (51.1)	0.023
Tumor size, *n* (%)				
< 1 cm	352	235(66.8)	117 (33.2)	0.54
1 − 3 cm	444	280 (63.1)	164 (36.9)	
> 3 cm	300	192 (64)	108 (36)	
Number of tumors, *n* (%)				
1	704	453 (64.35)	251 (35.65)	0.28
2 − 7	291	182 (62.54)	109(37.5)	
≥ 8	101	72 (71.3)	29 (28.7)	
Intravesical therapy, *n* (%)	472 (43.1)	294 (62.3)	178 (37.7)	0.18
Type of intravesical therapy, *n* (%)				
Early single instillation	145	89 (61.4)	56 (38.6)	0.60
Adjuvant chemotherapy	48	30 (62.5)	18 (37.5)	
Adjuvant BCG	279	175 (62.72)	104 (37.28)	
EAU risk group, *n* (%)				
Low-risk	78	53 (67.95)	25 (32.1)	0.76
Intermediate	519	336 (64.7)	183 (35.3)	
High-risk	499	318 (63.7)	181 (36.3)	

### Association with recurrence-free survival (RFS)

During follow-up, 462 (42.2%) patients developed pathologically confirmed disease recurrence, 177 (45.5%) patients with low AGR and 285 (40.3%) patients with normal AGR. The median time to recurrence was 27.5 months (IQR 8.3–68). Kaplan–Meier survival curves showed no significant difference in RFS between patients with low and normal serum AGR (supplementary Figure 1). There was no significant association between preoperative serum AGR and the risk of disease recurrence in univariable (HR 1.13, 95% CI 0.94–1.37, *P* = 0.19) and multivariable analyses (HR 1.10, 95% CI 0.91–1.33, *P* = 0.31) (Supplementary Table 1).

### Association with progression-free survival (PFS)

**Fig. 1:**
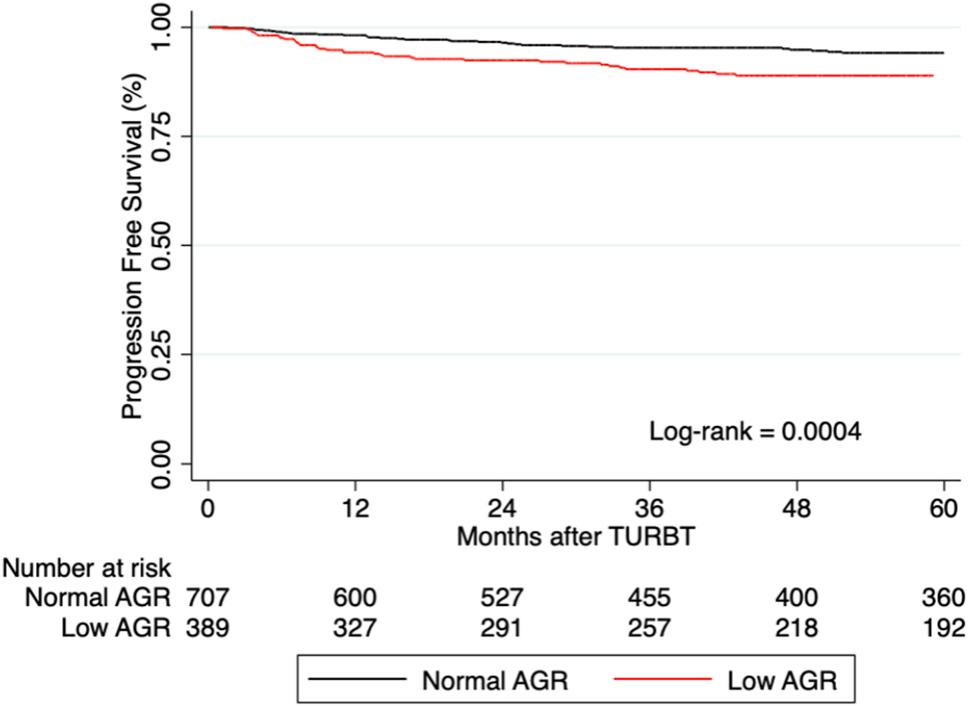
Progression-free survival in 1096 patients with non-muscle-invasive bladder cancer, stratified by preoperative serum albuminto-globulin ratio (AGR)

During follow-up, 101 (9.2%) patients experienced progression to MIBC; 52 (13.4%) patients with low AGR and 49 (6.9%) patients with normal AGR. The median time to progression was 25 months (IQR 8.8–68.3). Kaplan–Meier survival curves showed a significant difference in PFS between patients with low and normal serum AGR (Fig. 1). On a univariable analysis, low AGR was significantly associated with a higher risk of progression to MIBC (HR 1.99, 95% CI 1.35–2.94, *P* = 0.001). On multivariable analysis, preoperative AGR retained its independent association with PFS, after adjustment for the effects of established clinical and pathologic confounders (HR 1.81, 95% CI 1.22–2.68, *P* = 0.003). The inclusion of preoperative AGR marginally improved the discrimination of a base model that included established clinicopathologic features (C-index = 0.7354 vs. C-index = 0.7162) (Table 2).

**Table 2:** Univariable and multivariable Cox regression analyses for the prediction of progression-free survival in 1,096 patients with non-muscle-invasive bladder cancer

Progression free survival						
Variable	Univariable			Multivariable		
	HR	95% CI	*p*-value	HR	95% CI	*p*-value
Age	1.04	1.02–1.06	< 0.001	1.04	1.02–1.01	< 0.001
Gender						
Male	Reference	Reference	Reference			
Female	1.20	0.77–1.86	0.42			
Pathologic T stage						
pTa	Reference	Reference	Reference	Reference	Reference	Reference
pT1	1.57	1.06–2.32	0.02	0.48	0.23–0.99	0.050
Tumor grade						
G1	Reference	Reference	Reference	Reference	Reference	Reference
G2	2.56	1.19–5.52	0.02	2.14	0.98–4.66	0.057
G3	3.92	1.87–8.22	< 0.001	6.04	2.21–16.50	< 0.001
Concomitant CIS	1.37	0.52–3.38	0.49	0.79	0.31–1.99	0.61
Tumor size						
< 1 cm	Reference	Reference	Reference	Reference	Reference	Reference
1–3 cm	1.53	0.92–2.53	0.10	1.38	0.82–2.31	0.22
> 3 cm	1.80	1.08–3.01	0.025	1.40	0.82–2.37	0.22
Number of tumors						
Single	Reference	Reference	Reference	Reference	Reference	Reference
2–7	1.50	0.96–2.35	0.08	1.23	0.78–1.95	0.38
≥ 8	2.61	1.49–4.55	0.001	2.19	1.24–3.88	0.007
Intravesical therapy	1.13	0.76–1.68	0.55	0.94	0.61–1.45	0.78
AGR						
Normal	Reference	Reference	Reference	Reference	Reference	Reference
Low	1.99	1.35–2.94	0.001	1.81	1.22–2.68	0.003

### Subgroup analyses in patients based on their EAU risk group stratifications

In 499 patients who were classified in the EAU high-risk group, 187 (37.5%) and 62 (12.4%) patients experienced disease recurrence and progression, respectively. The median follow-up was 57 months. There was no statistically significant association between low AGR level and disease recurrence (HR 1.08; 95% CI 0.81–1.46, *P* = 0.59) or progression (HR 1.38; 95% CI 0.83–2.28, *P* = 0.22). Furthermore, no significant associations were found with recurrence or progression in the EAU low-risk group patients (Supplementary Table 2).

### Subgroup analyses in patients according to intravesical BCG therapy

Overall, 274 patients with EAU intermediate or high-risk group, who received adjuvant intravesical BCG therapy (induction and maintenance of at least 1 year). During a median follow-up of 59 months, 105 (38.3%) patients experienced disease recurrence, and 21 (7.7%) patients experienced disease progression to MIBC. Owing to the small sample size, the association with oncologic outcomes was tested in univariable analyses. Low preoperative AGR levels were not significantly associated with either disease recurrence (HR 1.12; 95% CI 0.76–1.66, *P* = 0.57) or progression (HR 1.65; 95% CI 0.69–3.89, *P* = 0.26) (Supplementary Table 2).

## Discussion

In recent years, there has been an increased awareness of the role of inflammation in relation to BCa development, progression and metastasis [15, 20–26]. Several systemic inflammatory markers have been evaluated in the literature and showed promising results [27, 28]. However, none of these factors have sufficient level of evidence to be implemented in the evaluation and management of BCa [11, 29]. The current prognostic tools for patients with NMIBC rely mainly on pathologic features of the tumor [30, 31]; integrating novel biomarkers can help improve the prognostic ability of these models [12, 13]. In a retrospective study of three systemic inflammatory markers (neutrophil-to-lymphocyte ratio (NLR), platelet-to-lymphocyte ratio (PLR), and lymphocyte-to-monocyte ratio (LMR), Cantiello et al. found that their combination in a predictive multivariable model can predict the risk of disease recurrence and progression in patients with high-risk NMIBC [22]. Similarly, D’Andrea et al. found that preoperative neutrophil-to-lymphocyte ratio is associated with both RFS and PFS in patients with NMIBC [20]. However, no previous study formally tested whether addition of AGR improves discrimination ability.

In this study, we investigated the association between preoperative AGR and NMIBC. We found that low preoperative AGR level predicts the risk of disease progression to MIBC. However, it did not predict the risk of disease recurrence. Only one study has previously evaluated the association of AGR with NMIBC. Niwa et al. found that low AGR was associated with a higher risk of both disease recurrence and progression in 364 patients with primary NMIBC [32]. In our study, we tested this lead further within a larger cohort of patients. We also performed subgroup analyses to identify the group of patients in whom this biomarker would likely be of most clinical benefit.

Patients with high-risk NMIBC are known to be subject to misclassification based on the current risk stratification tools. Risk assessment models for NMIBC, such as the EORTC and CUETO, have been criticized for the overestimation of the risk of disease progression, especially in patients with high-risk NMIBC [4, 33, 34]. In our study, we tested preoperative AGR in subgroups of patients based on their EAU risk groups; however, this biomarker failed to predict disease recurrence and progression in any of the risk groups. The reason underlying this finding can be multifold, such as the statistical power and the short follow-up.

Management of BCG unresponsive BCa remains one of the most challenging problems in urology, as these tumors have the highest risk of disease progression as well as metastasis [35–37]. Unfortunately, no current biomarker has sufficient evidence to identify the patients who are most likely to fail BCG therapy, which leads to delay in potential curative therapies (e.g., radical cystectomy) [37–39]. In our study, we further examined this biomarker in the subgroup of intermediate- and high-risk patients who were treated with BCG; here as well, there was no statistical association of preoperative AGR with disease recurrence or progression.

Albumin and globulin are major serum proteins and can reflect the systemic inflammatory response. Albumin modulates the systemic inflammatory reaction, as well as exert antioxidant effects. Albumin also plays an essential role in stabilizing cell growth and DNA replication. A low level of albumin has been a useful prognostic tool for various cancers. Globulin, on the other hand, increases with the accumulation of acute-phase proteins and immunoglobulins, which are reflective of an immunologic and inflammatory state [40, 41]. Emerging evidence has shown that AGR can be a useful predictive tool for cancer diagnosis and prognosis. Indeed, several studies reported that low serum AGR is correlated with worse outcomes in several cancers, such as gastric [16], colorectal [42], lung [43], and breast [44] cancers. However, this biomarker seems to have limited prognostic benefit in patients with NMIBC. Nevertheless, its association in our study with the risk of disease progression within the whole cohort can hint for a possible prognostic benefit. Probably further studies with better design (larger numbers, longer follow-up and more events) can evaluate this biomarker either alone, or in combination with other systemic inflammatory markers in this setting of BCa or, even, as a predictor to response of systemic therapies, such as immune checkpoint blockade.

The present study has several limitations, first, the retrospective design with its inherent selection bias. Second, because this is a multicentric study, different surgeons performed the TURB and the specimens were not reviewed by a central pathology. However, all participating centers are high-volume centers with experienced surgeons and genitourinary pathologists. Moreover, due to the multicentric nature of the study, repeat TURB was not routinely performed. Despite these limitations, we were able to provide a descriptive analysis of this biomarker in the different groups of NMIBC patients.

## Conclusion

Systemic inflammatory markers have a promising role in cancer prognosis. Low preoperative AGR was associated with a higher risk of disease progression in NMIBC patients but not with disease recurrence. Nevertheless, it failed to predict the risk of either recurrence or progression in patients based on their EAU risk groups and in BCG-treated patients. Further studies are needed to evaluate this marker either in its own or in combination with other systemic inflammatory markers to help build an optimal prognostic model for NMIBC and to guide therapeutic decision-making.

## Supplementary Information

Below is the link to the electronic supplementary material.

Supplementary file1 (PDF 200 KB)

Supplementary file2 (DOCX 17 KB)

Supplementary file3 (DOCX 20 KB)

## Abbreviations

AGR: Albumin-to-globulin ratio
NMIBC: Non-muscle-invasive bladder cancer
TURB: Transurethral resection of bladder tumor
IVT: Intravesical therapy
EAU: The European Association of Urology
MIBC: Muscle-invasive BCa
BCG: Bacillus Calmette–Guerin
BCa: Bladder cancer
RFS: Recurrence-free survival
PF: Progression free
