# Supplementary material for: Prognostic value of albumin to globulin ratio in non-muscle-invasive bladder cancer
Source: World J Urol. 2021 Jan 26;39(9):3345–52. doi: 10.1007/s00345-020-03586-1 (PMC8510920; doi:10.1007/s00345-020-03586-1)

Supplementary figure 1: Recurrence-free survival in 1096 patients with non−muscle-invasive bladder cancer, stratified by preoperative serum Albumin to-Globulin ratio (AGR).

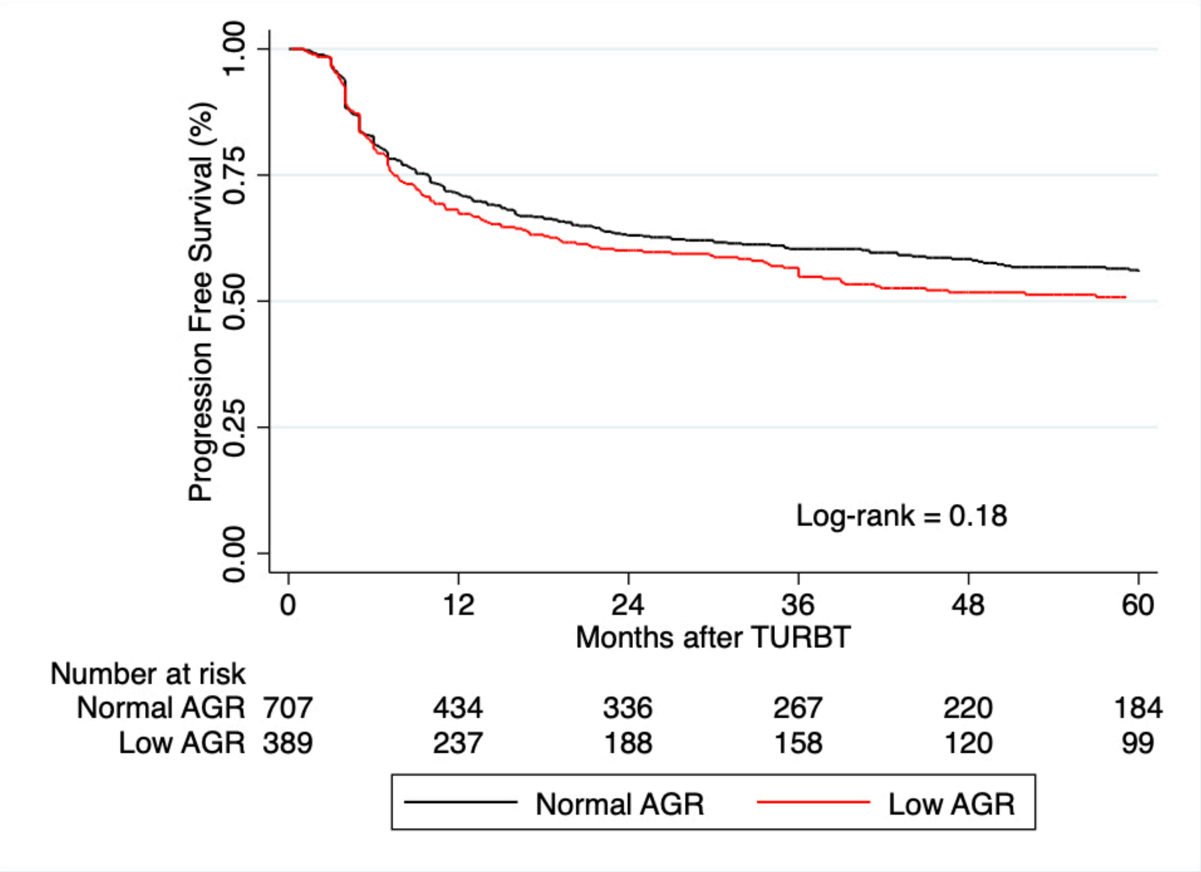

Supplement: Supplementary file 1 — Supplementary file1 (PDF 200 KB) [file 345_2020_3586_MOESM1_ESM.pdf]
