# Supplementary material for: Prognostic value of albumin to globulin ratio in non-muscle-invasive bladder cancer
Source: World J Urol. 2021 Jan 26;39(9):3345–52. doi: 10.1007/s00345-020-03586-1 (PMC8510920; doi:10.1007/s00345-020-03586-1)
Supplement: Supplementary file 2 — Supplementary file2 (DOCX 17 KB) [file 345_2020_3586_MOESM2_ESM.docx]

Supplementary table 1: Univariable and multivariable Cox regression analyses for the prediction of recurrence-free survival in 1,096 patients with non−muscle-invasive bladder cancer.

| **Recurrence Free Survival** | | | | | | | |
| --- | --- | --- | --- | --- | --- | --- | --- |
| **Variable** | **Univariable** | | |  | **Multivarilable** | | |
|  | **HR** | **95%CI** | **p-value** |  | **HR** | **95%CI** | **p-value** |
| **Age** | 1.03 | 1.02-1.03 | **<0.001** |  | 1.02 | 1.02-1.03 | **<0.001** |
| **Gender** |  |  |  |  |  |  |  |
| **male** | reference | reference | reference |  |  |  |  |
| **female** | 1.09 | 0.88-1.35 | 0.44 |  |  |  |  |
| **Pathologic T stage** |  |  |  |  |  |  |  |
| **pTa** | reference | reference | reference |  | reference | reference | reference |
| **pT1** | 0.71 | 0.59-0.86 | **<0.001** |  | 0.42 | 0.27-0.65 | **<0.001** |
| **Tumor grade** |  |  |  |  |  |  |  |
| **G1** | reference | reference | reference |  | reference | reference | reference |
| **G2** | 2.02 | 1.54-2.63 | **<0.001** |  | 1.58 | 1.20-2.08 | **0.001** |
| **G3** | 1.25 | 0.95-1.64 | 0.11 |  | 2.47 | 1.53-4.01 | **<0.001** |
| **Concommitant CIS** | 1.01 | 0.64-1.59 | 0.98 |  | 0.89 | 0.56-1.43 | 0.65 |
| **Tumor size** |  |  |  |  |  |  |  |
| **<1cm** | reference | reference | reference |  | reference | reference | reference |
| **1-3 cm** | 0.96 | 0.76-1.22 | 0.75 |  | 1.05 | 0.82-1.34 | 0.69 |
| **>3 cm** | 2.38 | 1.90-2.99 | **<0.001** |  | 2.30 | 1.82-2.91 | **<0.001** |
| **Number of tumors** |  |  |  |  |  |  |  |
| **single** | reference | reference | reference |  | reference | reference | reference |
| **2-7** | 1.51 | 1.23-1.84 | **<0.001** |  | 1.48 | 1.20-1.82 | **<0.001** |
| **≥8** | 1.01 | 0.72-1.43 | 0.95 |  | 1.08 | 0.76-1.54 | 0.66 |
| **Intravesical therapy** | 0.58 | 0.48-0.70 | **<0.001** |  | 0.61 | 0.49-0.75 | **<0.001** |
| **AGR** |  |  |  |  |  |  |  |
| **Normal** | reference | reference | reference |  | reference | reference | reference |
| **Low** | 1.13 | 0.94-1.37 | 0.19 |  | 1.10 | 0.91-1.33 | 0.31 |
