# Supplementary material for: Prognostic value of albumin to globulin ratio in non-muscle-invasive bladder cancer
Source: World J Urol. 2021 Jan 26;39(9):3345–52. doi: 10.1007/s00345-020-03586-1 (PMC8510920; doi:10.1007/s00345-020-03586-1)
Supplement: Supplementary file 3 — Supplementary file3 (DOCX 20 KB) [file 345_2020_3586_MOESM3_ESM.docx]

Supplementary table 2: Univariable Cox regression analyses for the prediction of progression-free survival and recurrence-free-survival in subgroups of patients with non−muscle-invasive bladder cancer based on: A) EAU low risk group, B) EAU high risk group, C) BCG treated EAU high risk group.

| 1. **EAU Low risk group** | | | | | | | |
| --- | --- | --- | --- | --- | --- | --- | --- |
| **Variable** | **Progression free survival** | | |  | **Recurrence free survival** | | |
|  | **HR** | **95%CI** | **p-value** |  | **HR** | **95%CI** | **p-value** |
| **Age** | 1.20 | 0.95-1.26 | 0.19 |  | 1.01 | 0.98-1.04 | 0.63 |
| **Intravesical therapy** | 8.89 | 0.36-222 | 0.18 |  | 0.32 | 0.07-1.39 | 0.13 |
| **AGR** |  |  |  |  |  |  |  |
| **Normal** | reference | reference | reference |  | reference | reference | reference |
| **Low** | 0.78 | 0.07-8.99 | 0.84 |  | 1.07 | 0.49-2.31 | 0.87 |
| 1. **EAU High risk group** | | | | | | | |
| **Variable** | **Progression free survival** | | |  | **Recurrence free survival** | | |
| **Age** | 1.04 | 1.01-1.07 | 0.001 |  | 1.02 | 1.01-1.04 | <0.001 |
| **Pathologic T stage** |  |  |  |  |  |  |  |
| **pTa** | reference | reference | reference |  | reference | reference | reference |
| **pT1** | 0.37 | 0.19-.72 | 0.003 |  | 0.43 | 0.28-.67 | <0.001 |
| **Concommitant CIS** | 0.55 | 0.21-1.43 | 0.22 |  | 0.73 | 0.43-1.23 | 0.24 |
| **Tumor size** |  |  |  |  |  |  |  |
| **<1cm** | reference | reference | reference |  | reference | reference | reference |
| **1-3 cm** | 1.33 | 0.67-2.62 | 0.42 |  | 1.13 | 0.74-1.71 | 0.56 |
| **>3 cm** | 1.46 | 0.74-2.88 | 0.27 |  | 2.80 | 1.91-4.10 | <0.001 |
| **Number of tumors** |  |  |  |  |  |  |  |
| **single** | reference | reference | reference |  | reference | reference | reference |
| **2-7** | 1.39 | 0.78-2.47 | 0.26 |  | 1.67 | 1.21-2.32 | 0.002 |
| **≥8** | 1.99 | 0.99-4.03 | 0.055 |  | 1.05 | 0.65-1.72 | 0.83 |
| **Intravesical therapy** | 0.78 | 0.47-1.31 | 0.35 |  | 0.59 | 0.44-.80 | 0.001 |
| **AGR** |  |  |  |  |  |  |  |
| **Normal** | reference | reference | reference |  | reference | reference | reference |
| **Low** | 1.38 | 0.83-2.28 | 0.22 |  | 1.08 | 0.81-1.46 | 0.59 |
| 1. **BCG treated EAU High risk group** | | | | | | | |
| **Variable** | **Progression free survival** | | |  | **Recurrence free survival** | | |
| **Age** | 1.04 | 0.99-1.09 | 0.097 |  | 1.02 | 0.99-1.04 | 0.14 |
| **Pathologic T stage** |  |  |  |  |  |  |  |
| **pTa** | reference | reference | reference |  | reference | reference | reference |
| **pT1** | 1.71 | 0.21-13.90 | 0.61 |  | 1.52 | 0.67-3.45 | 0.32 |
| **Tumor size** |  |  |  |  |  |  |  |
| **<1cm** | reference | reference | reference |  | reference | reference | reference |
| **1-3 cm** | 1.01 | 0.10-10.34 | 0.99 |  | 0.32 | 0.15-.69 | 0.004 |
| **>3 cm** | 4.06 | 0.48-34.59 | 0.20 |  | 1.19 | 0.60-2.36 | 0.61 |
| **Number of tumors** |  |  |  |  |  |  |  |
| **single** | reference | reference | reference |  | reference | reference | reference |
| **2-7** | 1.31 | 0.44-3.87 | 0.62 |  | 1.04 | 0.61-1.76 | 0.90 |
| **≥8** | 0.71 | 0.08-6.12 | 0.75 |  | 1.29 | 0.58-2.86 | 0.53 |
| **AGR** |  |  |  |  |  |  |  |
| **Normal** | reference | reference | reference |  | reference | reference | reference |
| **Low** | 1.02 | 0.35-2.93 | 0.97 |  | 1.29 | 0.78-2.14 | 0.31 |
